# IL-6 Impairs Vaccine Responses in Neonatal Mice

**DOI:** 10.3389/fimmu.2018.03049

**Published:** 2018-12-20

**Authors:** Jiyeon Yang, Jiro Sakai, Shafiuddin Siddiqui, Robert C. Lee, Derek D. C. Ireland, Daniela Verthelyi, Mustafa Akkoyunlu

**Affiliations:** ^1^Division of Bacterial Allergenic and Parasitic Diseases, Center for Biologics Evaluation and Research, US Food and Drug Administration, Silver Spring, MD, United States; ^2^Office of Biotechnology Products, Division of Biotechnology Review and Research III, Center for Drug Evaluation and Research, US Food and Drug Administration, Silver Spring, MD, United States

**Keywords:** neonates, follicular helper T cell, follicular regulatory helper T cell, IL-6, vaccine

## Abstract

The inability of infants to mount proper follicular helper T (T_FH_) cell response renders this age group susceptible to infectious diseases. Initial instruction of T cells by antigen presenting cells and subsequent differentiation into T_FH_ cells are controlled by T cell receptor signal strength, co-stimulatory molecules and cytokines such as IL-6 and IL-21. In immunized adults, IL-6 promotes T_FH_ development by increasing the expression of CXCR5 and the T_FH_ master transcription factor, B cell lymphoma 6. Underscoring the importance of IL-6 in T_FH_ generation, we found improved antibody responses accompanied by increased T_FH_ cells and decreased follicular regulatory helper T (T_FR_) cells, a Foxp3 expressing inhibitory CD4^+^ T cell occupying the germinal center (GC), when a tetanus toxoid conjugated pneumococcal polysaccharide type 14 vaccine was injected in adult mice together with IL-6. Paradoxically, in neonates IL-6 containing PPS14-TT vaccine suppressed the already impaired T_FH_ development and antibody responses in addition to increasing T_FR_ cell population. Supporting the diminished T_FH_ development, we detected lower frequency of phospho-STAT-3^+^ T_FH_ in immunized neonatal T cells after IL-6 stimulation than adult cells. Moreover, IL-6 induced more phospho-STAT-3^+^ T_FR_ in neonatal cells than adult cells. We also measured lower expression of IL-6R on T_FH_ cells and higher expression on T_FR_ cells in neonatal cells than adult cells, a possible explanation for the difference in IL-6 induced signaling in different age groups. Supporting the flow cytometry findings, microscopic examination revealed the localization of T_reg_ cells in the splenic interfollicular niches of immunized adult mice compared to splenic follicles in neonatal mice. In addition to the limitations in the formation of IL-21 producing T_FH_ cells, neonatal mice GC B cells also expressed lower levels of IL-21R in comparison to the adult mice cells. These findings point to diminished IL-6 activity on neonatal T_FH_ cells as an underlying mechanism of the increased T_FR:_ T_FH_ ratio in immunized neonatal mice.

## Introduction

Diminished host response to vaccines during the neonatal term is well-established in humans and in animal models (1). Critical differences in the innate and adaptive immune systems of infants and adults provide clues to reasons for the delay in the development of protective immune response in vaccinated infants (2, 3). Reduced expression of the TNF family receptor transmembrane activator and calcium modulator and cyclophilin ligand interactor (TACI) on neonatal B cells is likely responsible for the unresponsiveness to T cell-independent polysaccharide vaccines in this age group (4). Vaccines that require T-cell help for the production of antibodies need to be administered three to four times during the first 15 months of life in order to afford protection (1). In adults, the encounter of naïve CD4^+^ T cells with peptides on dendritic cells (DC) located in the T cell zone of lymphoid organs leads to the expression of CXCR5 on T cells and their relocation to germinal centers (GC) (5). Further interactions between CD4^+^ T cells and GC B cells promote the differentiation of T cells into follicular helper T (T_FH_) cells that express the transcription factor B cell lymphoma 6 (Bcl6) and secrete IL-21. Differentiated T_FH_ cells, which express varying degrees of programmed cell death protein 1 (PD-1), enhance their interaction with GC B cells through cell surface molecules such as inducible T-cell co-stimulator (ICOS) and CD40L. IL-21 secreted from T_FH_ cells not only amplifies T_FH_ differentiation in an autocrine fashion but also acts on GC B cells for the development of plasma cells and memory B cells (6, 7). The success of a T cell dependent (TD) antibody-response requires tight regulation of T_FH_ development and kinetics, as excessive T_FH_ response may result in the development of lower-affinity antibodies and plasma cells with shorter life span (8, 9). Recently identified, Foxp3-expressing regulatory T_FH_ (T_FR_) cells are especially critical in controlling T_FH_ cell development by a yet-to-be-identified mechanism (10, 11). Ultimately, the ratio of T_FR_ to T_FH_ (T_FR:_ T_FH_) cells in GC can help predict the influence of T_FR_ cells on antibody responses (12–14).

Studies in mice indicate that T_FH_ development and GC reaction is severely impaired in vaccinated neonates (15, 16). As suggested by adoptive transfer experiments, both, host environmental factors and T cell intrinsic mechanisms appear to contribute to the limited T_FH_ cell-development in neonates (15). Moreover, high T_FR:_ T_FH_ ratio in the GC of immunized neonatal mice suggests that the presence of high numbers of T_FR_ cells may be adversely influencing the GC environment in immunized neonates (13, 14, 17, 18). In adults, vaccination or infection results in a dynamic change in the number of T_FH_ and T_FR_ cells occupying GC in lymphoid tissues (14, 19). Coinciding with the decrease in T_FR_ cell-proportion, the increase in T_FH_ cell-proportion allows for the successful interaction of T_FH_ cells with GC B cells. The increase in T_FR_ cell numbers precedes the resolution of GC reaction, therefore GC reaction appears to be also orchestrated by T_FR_ cells. Better understanding of the cellular and molecular mechanisms responsible for the altered T_FH_ development and high T_FR:_ T_FH_ ratio in immunized neonatal mice will be needed to devise improved vaccines for this age group.

In this study, we investigated the kinetics of T_FH_ and T_FR_ development in neonatal and adult mice and confirmed that a high T_FR:_ T_FH_ ratio persists throughout the immune response in immunized neonatal mice. In adults, IL-6 promotes the activation and differentiation of CD4^+^ cells during the interaction of naïve CD4^+^ T cells with DCs located in the T-cell zone of lymphoid tissues (20). We found that inclusion of IL-6 to a pneumococcal type 14 polysaccharide-tetanus toxoid (PPS14-TT) conjugate vaccine resulted in opposite outcome in neonatal and adult mice. As shown previously, IL-6 increased antibody responses to the co-administered vaccine (21) and augmented T_FH_ development while suppressing T_FR_ cell response. In contrast, IL-6 suppressed neonatal mice antibody responses and T_FH_ generation. Moreover, IL-6 increased the T_FR:_ T_FH_ ratio in neonatal mice. In support of the diminished antibody responses and increased T_FR:_ T_FH_ ratio, IL-6 induced STAT3 phosphorylation was more in T_FR_ cells than T_FH_ cells in immunized neonatal mice. These observations advance our understanding of the mechanisms of suboptimal TD antibody response observed in neonates and can help to improve vaccines intended for neonates.

## Materials and Methods

### Mice

Wild-type mice with a C57BL/6 genetic background were purchased from Jackson Laboratory, bred, and kept in pathogen-free animal facilities in accordance with FDA Veterinary Services guidelines. For adult mouse experiments, 6 to 10 week old female mice were used. For neonatal mouse experiments, 5 day-old mice were used in immunization studies and 7 day-old mice were used for *in vitro* differentiation studies. All animal procedures were approved by FDA Institutional Animal Care and Use Committee (Protocol 2002-31).

### Immunization

Adult mice were immunized intraperitoneal (i.p.) with 2 × 10^8^ sheep red blood cells (SRBC) and neonatal mice with 0.5 × 10^8^ SRBC (Rockland Immunochemicals, Pottstown, PA). PPS14-TT vaccine was manufactured as described (22). PPS14-TT vaccine (1 μg per adult and 0.2 μg per neonatal mouse) together with recombinant IL-6 (500 ng/adult, 100 ng/neonate, from R&D Systems) was emulsified with aluminum hydroxide [Al(OH)_3_] (Thermo Fisher Scientific, Waltham, MA), 1/3 of injection volume. Intraperitoneal injection volumes were 150 μl for adult and 30 μl for neonatal mouse.

### Sorting and NCounter Nanostring

Single-cell suspensions of splenocytes were diluted in PBS supplemented with 1% FBS and 1 mM EDTA. Follicular T cells and non-follicular T cells were isolated from CD4^+^ cells after enriching with a magnetic positive selection kit (Miltenyi Biotec, Bergisch Gladbach, Germany). CD4^+^ enriched cells were stained and sorted as follows: CD4^+^CXCR5^+^PD-1^+^ follicular T cells and CD4^+^CXCR5^−^PD-1^−^ non-follicular T cells. For B cell isolation, flow-through from CD4^+^ selection was subjected to positive selection with CD19 beads (Miltenyi Biotec). CD19^+^-enriched cells were stained and sorted as follows: B220^+^GL7^+^FAS^+^ GC B cells and B220^+^GL7^−^FAS^−^ non-GC B cells. Gene expression analysis of sorted cells were performed on nCounter Immunology Panels. Data have been deposited into the GEO series database (GSE117648).

### Ingenuity Pathway Analysis

IL-21 or IL-4 activated/inhibited genes on GC B cells were predicted by upstream analysis in Ingenuity Pathway Analysis (IPA, Ingenuity Systems, www.ingenuity.com). The 69 differentially expressed genes (*p* < 0.05, >1.5-fold) were uploaded into IPA for analysis.

### Antibody for FACS Analysis

Single-cell suspensions were prepared from splenocytes. To stain dead cells, the suspensions were incubated with fixable efluor 780 (Affymatrix, Santa Clara, CA) diluted at 1:1,000 dilution in PBS for 10 min at room temperature. Cells were washed and stained using FACS buffer containing 2% FBS, 0.5M EDTA in PBS. The following antibodies were used for surface staining at room temperature: α-CD4 (BD Biosciences, 1:200, GK1.55), α-PD-1 (BD Biosciences, 29F.1A12), α-CXCR5 (biotin, BD Biosciences, 2G8; BioLegend, L138D7), α-GL7 (BD Biosciences, GL-7), α-FAS (BD Biosciences, J02), α-CD25 (BioLegend, San Diego, CA, PC61), α-IL-6Rα (biotin, Biolegend, D7715A7), GP130 (R&D system, Q6PDI9), α-IL-21R (biotin, eBioscience, eBioA9), α-ICOSL (biotin, HK5.3, BioLegend), CD19 (6D5, Biolegend), CD23 (B3B4, eBioscience), Bcl6 (7D1, Biologend). To detect biotinylated CXCR5, IL-6Rα, IL-21R, and ICOSL antibodies, cells were further incubated with streptavidin-BV-421 (BD Bioscience, 1:500) for 15 min at room temperature. For intracellular staining, samples were fixed with the Foxp3 Fix/Perm buffer set by following the manufacturer's instructions (eBioscience). Samples were then intracellularly stained with α-Foxp3 (BioLegend, 150D, 1:100) antibody. Flow cytometry data were acquired on LSRII flow cytometer (BD Biosciences) and analyzed using the FlowJo software v10 (Tree Star, Inc., Ashland, OR).

### Intracellular Cytokine FACS Analysis

Single-cell suspensions of splenocytes were stimulated with PMA (1 μg/ml) and ionomycin (1 μg/ml) (both from Sigma-Aldrich,) in the presence of GolgiStop™ (BD Biosciences, 1:1,000) at 37°C for 4 h. Cells were incubated with antibody for CD4, and PD-1 at 4°C, then were fixed and permeabilized with Foxp3 Fix/Perm buffer set (eBioscience) and incubated with antibody for IL-2 (BD Biosciences, JES6-5H4), IL-4 (BD Biosciences, 11B11), IL-10 (eBioscience, JES5-16E3), and IFNγ (BD Biosciences, XMG1.2). For IL-21 staining, cells were incubated with IL-21 R/Fc chimera (R&D Systems) for 1 h, washed and stained with PE-labeled affinity-purified F(ab') α-human IgG Fc Region antibody (R&D Systems) for 30 min.

### Phospho-STAT3 FACS Analysis

Single-cell suspensions were incubated in culture media supplemented with 10% FBS, alone or with mouse recombinant IL-6 (R&D Systems, 100 ng/ml), or IL-21 (R&D Systems, 50 ng/ml) for 15 min at 36°C. After washing with FACS buffer containing 1% FBS with 1mM EDTA, cells were first fixed with BD Cytofix fixation buffer for 10 min at 36°C, and then permeabilized with pre-chilled BD Phosphoflow buffer III for 10 min at 4°C. Cell surface antibodies and α-phospho-STAT3 (Cell Signaling, Y705 [D3A7], 1:200 dilution) antibody were incubated together in FACS buffer for 30 min at room temperature. For further phospho-STAT3 detection, a goat anti-rabbit-AF488 secondary [Life Technologies (Thermo Fisher Scientific), 1:500 dilution] antibody was incubated for 15 min at room temperature. For detection of T_FR_ cells, cells were washed and Foxp3 antibody was added to the permeabilization buffer (eBioscience) for 30 min at room temperature or 2 h at 4°C.

### Follicular Bcl6^+^ B Cell Generation

Mouse B cells were purified from splenocytes by negative selection (Miltenyi Biotec). Cells were cultured in media alone, with IL-21 (10 ng/ml, R&D Systems), IL-21 with CD40L (1 μg/ml, PeproTech, Rocky Hill, NJ) or with CpG oligonucleotide (25 μg/ml) (23) for 72 h. Stimulated cells were analyzed in flow cytometry for the measurement of Bcl6 expression on CD19^+^CD23^+^ population.

### Immunofluorescence Confocal Microscopy

OCT-embedded spleens were snap-frozen by floating on liquid nitrogen-cooled isopentane. Frozen tissues were cut into 10 μm sections using a cryostat and mounted on poly-lysine-coated slides. Sections were allowed to air dry at room temperature for 10 min and fixed with pre-cooled Acetone and Methanol (1:1 vol) for 10 min, followed by washing three times with PBS containing 0.5% Tween-20. Sections were first blocked with 5% donkey or goat serum for 1 h, and then stained overnight at 4°C with α-B220 (Rat IgG, 1:50, eBiosicence), α-GL7 (Rat IgM, 1:50, BioLegend), α-Foxp3 (rabbit IgG, 1:200, Abcam), α-Podoplanin (goat IgG, 1:100, R&D Systems) CD4-biotin (1:50, BioLegend) antibodies. Following three washing steps with PBS containing 0.5% Tween-20, sections were stained for 1 h at room temperature with the following secondary antibodies: goat or donkey α-rat IgG-AF488, goat α-rat IgM-AF647, goat or donkey α-rabbit IgG-AF586, donkey anti-goat IgG-AF647 (1:200, Jackson ImmunoResearch Laboratories), and Streptavidin-AF405 (1:100, Life Technologies). Samples were imaged on Zeiss 780 laser scanning microscope using multichannel frame scans. The z-stacks were converted to projections using ZEN Black. The ZEN tile scan function was used to stitch individual 10x images together.

### Measurement of Antibody Titers Against PPS14

For antibody measurement, 96-well plates were coated with purified PPS14 (ATCC, Manassas, Virginia) at 10 μg/ml in PBS (pH of 7) for 2 h at room temperature and then blocked for 1 h with 5 % neonatal calf serum (Sigma) in PBS. Serum samples (1:100 dilution for adult or 1:20 dilution for neonates) were serially diluted and 100 μl of diluted samples were transferred on coated plates for overnight incubation. After washing, wells were incubated with horseradish peroxidase-conjugated goat anti-mouse IgG-Fc or IgA antibody (Bethyl Laboratories) for 3 h at room temperature and detected by ECL substrate.

### Statistical Analysis

Unpaired student's *t*-test was used for all comparisons; data represented as mean +/− SEM are shown. *P*-values < 0.05 were considered statistically significant.

## Results

### Restricted T_FH_ Generation Is Accompanied by Severely Reduced IL-21 Production in Neonatal Mice

**Figure 1:**
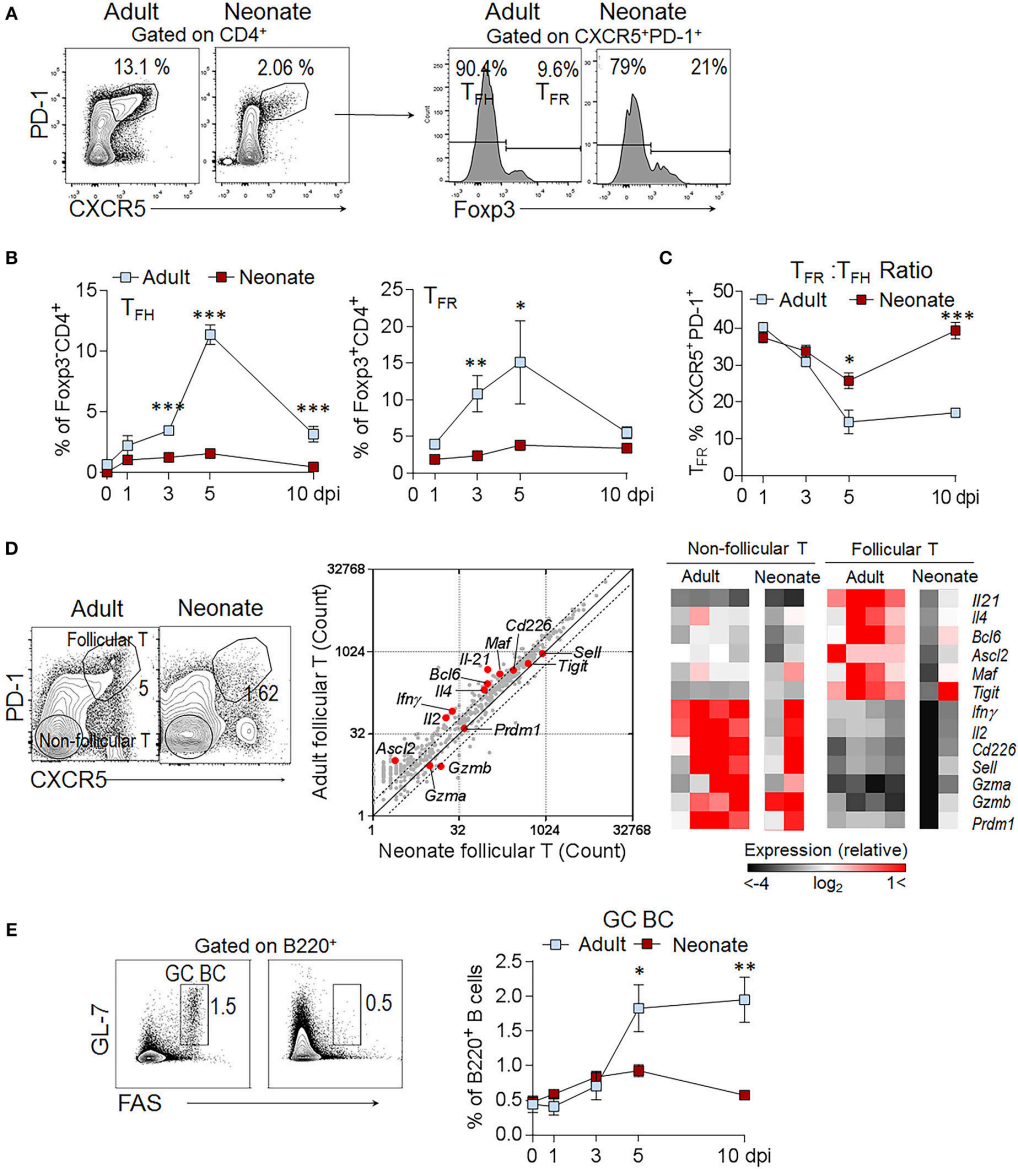
High T_FR_: T_FH_ ratio persists throughout the immune response in immunized neonatal mice. Adult (6- to 10-week-old) and neonatal (5 day-old) mice were immunized i.p. with SRBC. Kinetics of splenic T_FH_ and T_FR_ cell generation was assessed in flow cytometry. **(A)** Representative dot plots depict the percentage of T_FH_ (CD4^+^CXCR5^+^PD-1^+^Foxp3^−^) and T_FR_ (CD4^+^CXCR5^+^PD-1^+^Foxp3^+^) cells pre-gated on CD4^+^ cells at 5 dpi. **(B)** Mean percentage of T_FH_ cells among Foxp3^−^CD4^+^ and T_FR_ cells among Foxp3^+^CD4^+^ cells are plotted (*n* = 4). **(C)** The ratio of T_FR_ to T_FH_ cells (T_FR_: T_FH_) are plotted (*n* = 4). **(D)** Analysis of gene expression profile in sorted follicular and non-follicular T cell populations. Flow cytometry gating strategies for follicular (CD4^+^CXCR5^+^PD-1^+^) and non-follicular (CD4^+^CXCR5^−^PD-1^−^) T cells are shown at 10 dpi. Expression of 547 immunology-related genes in neonatal vs. adult follicular T cells (gray) is plotted. Each plot displays average mRNA gene-counts and fold change. The dashed lines represent a 2-fold change. Heat maps represent the relative expression of T_FH_ associated genes. Red represents relatively high expression, whereas black represents relatively low expression. Each column represents one replicate pooled from three adult or eight neonatal mice. **(E)** Representative dot plots depict the GC (GL-7^+^FAS^+^) cells pre-gated on B220^+^ cells at 7 dpi. Mean percentages of GC B cells are plotted (*n* = 4). One out of three experiments with similar results is shown. Error bar, s.e.m.. *p < 0.05, **p < 0.01, ***p < 0.001 depict comparisons between adult and neonatal mice.

Reduced T_FH_ cell generation in immunized neonatal mice is well established (16). Our sheep red blood cell (SRBC) immunization experiments confirmed the significantly lower percentage of T_FH_ (CD4^+^Foxp3^−^CXCR5^+^PD-1^+^) cells in neonatal mice as compared to adult mice throughout the course of host response (Figures 1A,B). Supporting the flow cytometry data, the expression of T_FH_ cell differentiation-promoting genes (*Il21, Il4, Bcl6, Ascl2, and Maf)* were not as pronounced in neonatal T_FH_ cells as in adult T_FH_ cells (Figure 1D). IL-21 expression by T_FH_ cells is especially important for TD antibody responses; because IL-21 not only helps expand T_FH_ cells, but also acts on GC B cells to induce the generation of memory B cells and plasma cells (7). Previous studies reported ablated T_FH_ generation accompanied by low IL-21 expression in neonatal mice (15, 16). Confirming the gene array results, we measured lower IL-21 in addition to reduced IFNγ production in neonatal follicular T cells than adult cells without a significant difference in IL-4 and IL-10 production following immunization (Supplementary Figure 1). Although the percentages of the inhibitory CD4^+^Foxp3^+^CXCR5^+^PD-1^+^ T_FR_ cells in neonates were less than those in adult mice, the T_FR:_ T_FH_ ratio was higher on 5 and 10 days post immunization (dpi) in neonatal mice as compared to adult mice (Figures 1A–C). Similarly, confirming previous reports (15, 16), the generation of GC B cells was limited in neonates compared with adult mice (Figure 1E).

### Co-administration of IL-6 With PPS14-TT Vaccine Suppresses T_FH_ Cell Generation and Dampens Antibody Responses in Neonatal Mouse

**Figure 2:**
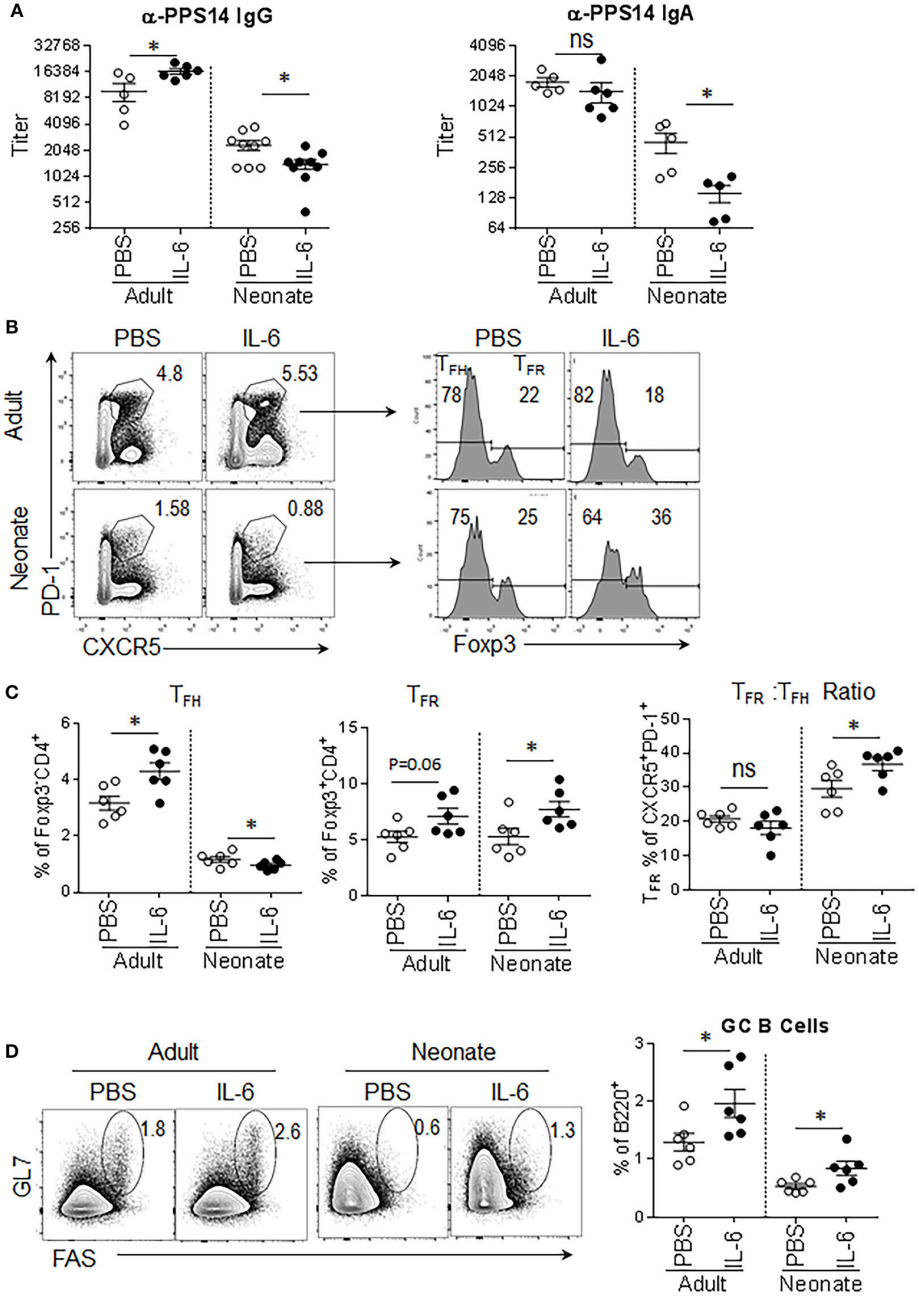
Antibody responses to PPS14-TT vaccine are diminished when IL-6 is co-injected. Adult (6–10 week-old) and neonatal (5 day-old) mice were immunized i.p. with PPS14-TT alone or with IL-6 (500 ng/adult, 100 ng/neonate) and splenocytes were analyzed in flow cytometry at 7 dpi. **(A)** Serum anti-PPS14 IgG and IgA titers were determined in ELISA 6 weeks after immunization (*n* = 6–9). **(B)** Representative dot plots depict the percentage of T_FH_ (CD4^+^CXCR5^+^PD-1^+^Foxp3^−^) and T_FR_ (CD4^+^CXCR5^+^PD-1^+^Foxp3^+^) cells. **(C)** Mean percentages of T_FH_ cells among Foxp3^−^CD4^+^ cells and T_FR_ cells among Foxp3^+^CD4^+^ cells are shown. The ratio of T_FR_ to T_FH_ cells (T_FR_: T_FH_) are plotted (*n* = 6). **(D)** Representative dot plots depict mean percentage of GC B (GL-7^+^FAS^+^) cells. Plots were pre-gated on B220^+^ cells (*n* = 6). One out of two experiments with similar results is shown. Error bar, s.e.m.. *p < 0.05.

Next, we focused on the factors that govern the production of IL-21. In TD antibody responses, IL-6 secreted by the DCs during the antigen presentation triggers IL-21 expression in naïve CD4^+^ T cells (21, 24–26) and IL-21 produced by these cells further promotes IL-21 production in an autocrine fashion (6, 7). Highlighting the importance of IL-6 in TD antibody responses, in adult mice, the injection of IL-6 with antigen amplifies the antigen-specific antibody responses in an IL-21 dependent manner (21). To assess whether the supply of IL-6 can stimulate IL-21 production and improve antibody responses in neonatal mouse also, we immunized adult and neonatal mice with the PPS14-TT vaccine combined with IL-6. Confirming previous reports, adult mice immunized with the PPS14-TT vaccine and IL-6 elicited higher IgG antibodies against PPS14 than did mice immunized with vaccine alone (Figure 2A). Surprisingly, IL-6 not only did not improve antibody responses, it further dampened anti-PPS14 IgG and IgA antibody levels compared to mice immunized with the vaccine alone (Figure 2A). Moreover, unlike in adult mice, where an increase in T_FH_ cell frequency accompanied the elevation of antibody response, in neonatal mice IL-6 containing vaccine decreased the percentage of T_FH_ cells among CD4^+^Foxp3^−^ population compared to vaccine without IL-6 (Figures 2B,C). In addition to the reduction in T_FH_ cell population, IL-6 receiving neonatal mice manifested a significant increase in the percentage of T_FR_ cells among the T_reg_ and CD4^+^CXCR5^+^PD-1^+^ population. Interestingly, IL-6-containing vaccine significantly increased GC B cells in adult and neonatal mice despite its divergent effect on T_FH_ cells in the two age groups (Figure 2D). Although T_FH_ formation influences GC development and vice versa (27), IL-6 also can directly stimulate B cells (28). Thus, the expansion of GC B cells in neonatal mice could be a result of a direct effect of IL-6 on B cells.

### IL-6 Signaling Is Impaired in Neonatal T_FH_ Cells

**Figure 3:**
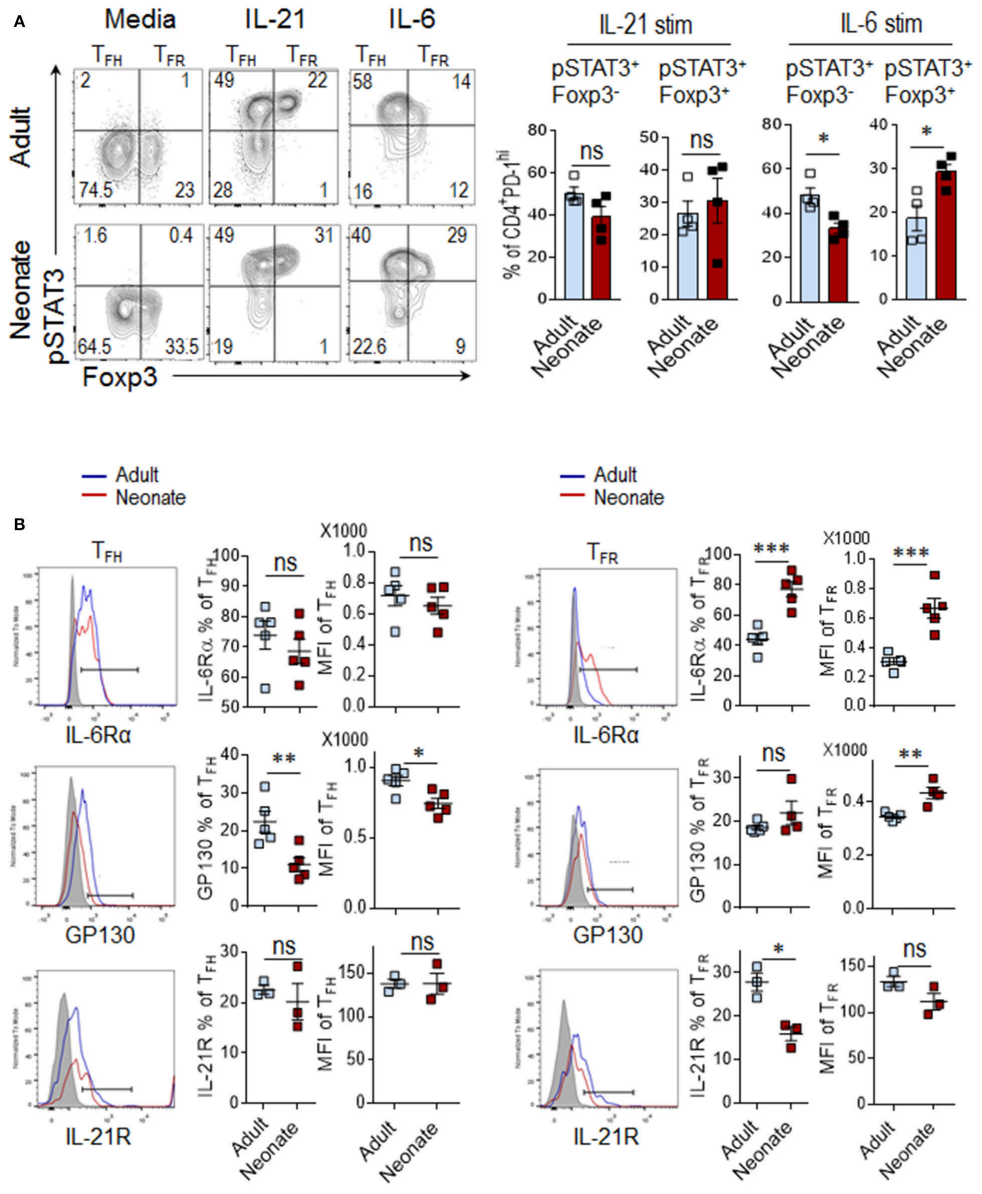
IL-6 signaling preferentially induces T_FR_ differentiation in neonatal mice. Adult (6 to 10-week-old) and neonatal (5 day-old) mice were immunized i.p. with SRBC, and STAT3 activity in splenic CD4^+^ cells was analyzed in flow cytometry at 5 dpi. **(A)** For phospho-STAT3 activity measurement, splenocytes were stimulated with IL-21 (50 ng/ml) or IL-6 (100 ng/ml) for 15 min, followed by phospho-flow staining. Plots showing the percentage of pSTAT3^+^ cells among Foxp3^+^ or Foxp3^−^ populations were pre-gated on CD4^+^PD-1^hi^ cells (Supplementary Figure 2). **(B)** Analysis of IL-6Rα, GP130, and IL-21R expression on T_FH_ and T_FR_ populations. Cells in histograms were pre-gated on CD4^+^CXC5^+^PD-1^+^Foxp3^−^ T_FH_ and CD4^+^CXC5^+^PD-1^+^Foxp3^+^ T_FR_ cells as shown in Figure 1A. One out of three experiments with similar results is shown. Error bar, s.e.m.. *p < 0.05, **p < 0.01, ***p < 0.001. Stim, stimulation.

The inability of IL-6 to improve antibody responses in neonatal mouse prompted us to assess the activity of IL-6 on neonatal CD4^+^ T cells. Both IL-21 and IL-6 converge on the STAT3 pathway for the differentiation and/or maintenance of T_FH_ cells (26, 29). To investigate the contribution of IL-21 and IL-6 on T_FH_ differentiation, we measured the frequency of phospho-STAT3^+^ cells among CD4^+^PD-1^hi^Foxp3^−^ (T_FH_) population (see Supplementary Figure 2 for CD4^+^PD-1^hi^ gating strategy) after *in vitro* stimulation of splenocytes from SRBC immunized neonatal and adult mice (5 dpi) with IL-21 or IL-6. We detected substantial and comparable increases in phospho-STAT3^+^ in T_FH_ (Foxp3^−^) cells in response to IL-21 in both adult and neonatal mice (Figure 3A). Next, we analyzed STAT3 activity in CD4^+^PD-1^hi^Foxp3^+^ T_FR_ cells because in addition to directly acting on T_FH_ cells, IL-21 can also enhance T_FH_ generation by restricting the T_FR_ cell proliferation through its inhibitory effect on T_FR_ cell IL-2 responses (30). We found no difference in the frequency of phospho-STAT3^+^ T_FR_ (Foxp3^+^) cells between adult and neonatal cells in response to IL-21 (Figure 3A). In agreement with the comparable IL-21 activity on T_FH_ and T_FR_ cells between neonatal and adult cells, the expression of IL-21R was not different on these cells between the two age groups (Figure 3B). In contrast to IL-21, IL-6 activity was markedly different between the neonatal and adult cells. IL-6 triggered significantly lower percentage of phospho-STAT3^+^ T_FH_ cells in neonates than in adults (Figure 3A). The activity of IL-6 is dependent on the homodimer formation of IL-6Rα and GP130 receptor complexes (28) and the level of IL-6R expression correlates with IL-6 induced STAT3 phosphorylation (31). Interestingly, we found significantly lower frequency and expression of GP130, the component that is responsible for STAT3 phosphorylation (32), in neonatal T_FH_ cells than adult cells (Figure 3B).

Although a direct contribution of IL-6 to T_FR_ cell development has not been demonstrated, the fact that IL-6 produced by extrafollicular DCs support the T_FH_ generation (6), and STAT3 has recently been shown to be important in the differentiation of T_FR_ cells (33), a similar effect on T_FR_ cell activation is conceivable. Our analysis of T_FR_ cells indicated that in sharp contrast to T_FH_ cells, IL-6 induced higher frequency of phospho-STAT3^+^ in neonatal T_FR_ cells than in adult cells (Figure 3A). A plausible explanation for the divergent effect of IL-6 on neonatal and adult T_FR_ cells emerged from IL-6Rα expression analysis. The frequency and the level of neonatal IL-6Rα expression were significantly higher in T_FR_ cells as compared to those of adult mice (Figure 3B). Neonatal T_FR_ cells also expressed higher levels of the IL-6R subunit β, GP130, although the frequency of cells expressing GP130 was not different between the age groups (Figure 3B). Thus, while IL-21 signaling in neonatal T_FH_ cells is intact, compared to adult cells, IL-6 induces limited STAT3 phosphorylation in neonatal T_FH_ cells, a likely consequence of diminished expression of GP130 component of IL-6R on these cells. At the same time, neonatal T_FR_ cells express higher levels of IL-6R and signal more than their adult counterparts in response to IL-6.

### T_reg_ Cells Occupy the Splenic Interfollicular Niches in Adults, While Residing in Follicles in Neonatal Spleens

**Figure 4:**
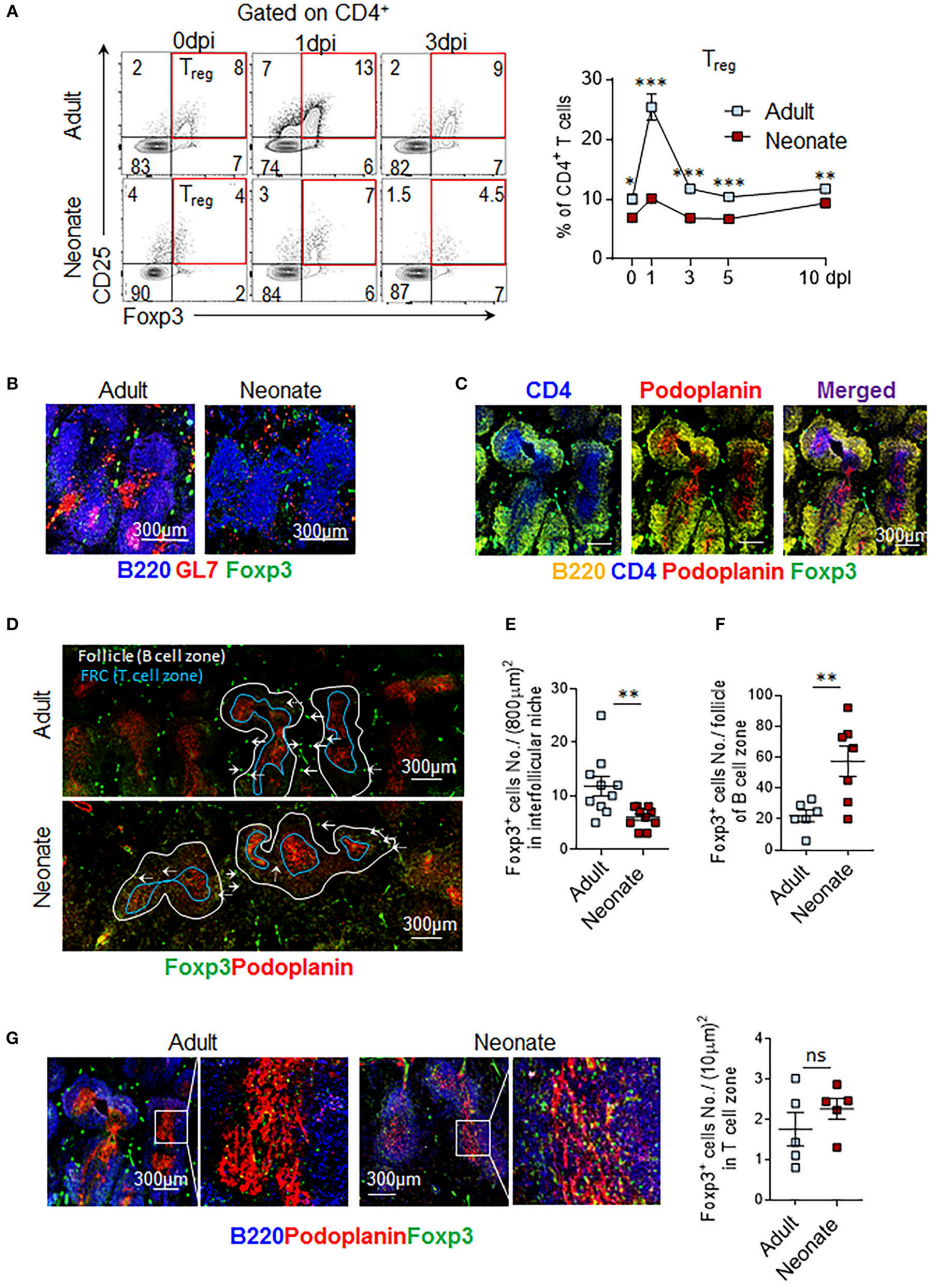
T_reg_ cells populate interfollicular niches in adult, and follicles in neonatal spleen. Adult (6 to 10-week-old) and neonatal (5 day-old) mice were immunized i.p. with SRBC. **(A)** Representative dot plots of T_reg_ (CD4^+^CD25^+^Foxp3^+^) cells from 0, 1, and 3 dpi are shown. Mean percentages of T_reg_ cells among CD4^+^ cells are plotted (*n* = 4). Splenic GC, FRC, and Foxp3^+^ cells were examined by microscopy at 14 dpi **(B-G)**. **(B)** B220^+^GL7^+^ GCs and GC B cells (merged pink color) are shown. **(C)** Representative image of adult T cell-zones, defined as areas where CD4 and FRC are co-localized, is shown. Podoplanin is used as a marker of FRC. **(D-G)** Distribution of T_reg_ cells in adult and neonatal spleen is depicted. **(E)** Follicles (B220^+^ B cell area) were outlined using white and blue lines. Blue line demarks T-B border. Foxp3^+^ cells in interfollicular niches (solid arrows) **(D)** or within follicle (dotted arrows) **(F)** were quantified in half spleen and means of cells in each compartment were plotted. **(G)** Foxp3^+^ cells in T cell-zone within FRC area were captured in box (left) and enlarged (right). Each dot represents follicles from four mice, representative of two experiments. Magnification 10x. FRC, follicular reticular cell. *p < 0.05, **p < 0.01, ***p < 0.001 depict comparisons between adult and neonatal mice.

Given the importance of IL-6 activity in the formation of T_FH_ cells, the limitations in IL-6 induced STAT3 signaling in neonatal T_FH_ cells likely play a significant role in the inability of IL-6 to promote T_FH_ development and antibody responses in neonatal mice. However, the fact that IL-6 triggered higher phospho-STAT3 in neonatal T_FR_ cells than adult cells also suggests that the persistence of T_FR_ cells in neonatal cells may be further contributing to ablated T_FH_ response by increasing the T_FR:_ T_FH_ ratio. Persistence of a high T__FR_:_ T_FH_ ratio in neonatal mice is especially noteworthy because in adult mice T_FR:_ T_FH_ ratio declined despite having significantly higher percentage of splenic precursor T_reg_ cells than neonatal mice following immunization (Figure 4A). One possible explanation for this paradoxical result is the preferential commitment of CD25^+^ and Foxp3-expressing neonatal CD4^+^ T cells to T_FR_ cells.

To further examine this hypothesis, we compared the distribution of Foxp3^+^ cells in histological sections of immunized adult and neonatal mouse spleens. Multiple GCs were formed in B cell follicles only in adult mouse spleens (Figure 4B). As reported previously, neonatal mice failed to form GCs (15, 16). We next quantified Foxp3^+^ cells throughout the splenic sections to determine T_FR_ and T_reg_ cells based on their locations. As reported previously for lymph nodes, Foxp3^+^ cells were found in three different splenic locations; T cell-zone, follicle, and interfollicular niche (34) (Figures 4B,C). Foxp3^+^ cells in follicles were considered T_FR_ cells, whereas Foxp3^+^ cells in the T cell zone and interfollicular niches were considered T_reg_ cells. Fibroblast reticular cells (FRC) are specialized stromal cells localized in the T cell zone where they segregate T cells from B cells (35). Follicles and T cell zones were outlined by staining B220 and podoplanin (a marker of FRC), respectively (Figure 4C). T_reg_ cell counts in interfollicular niches were significantly lower in neonatal mice as compared to adult mice (Figures 4D,E). In contrast, neonatal mice had more T_FR_ cells within B cell follicles than did adult mice (Figures 4D,F). Consistently higher numbers of Foxp3^+^ cells per follicle count, but lower Foxp3^+^ cells in interfollicular niches (Figures 4D–F) in histological analysis support the higher T_FR:_ T_FH_ ratio finding, despite lower T_reg_ expansion in immunized neonatal mice (Figure 4A). Occupancy of T-cell-zones with T_reg_ cells was comparable between neonatal and adult mice (Figure 4G). These findings suggest that the lymphoid environment controlling the expansion of T_reg_ cells and their differentiation into T_FR_ cells are different in neonatal and adult secondary lymphoid tissues.

### The CD25^−^ T_FR_ Population Expands in Immunized Neonatal Mice

**Figure 5:**
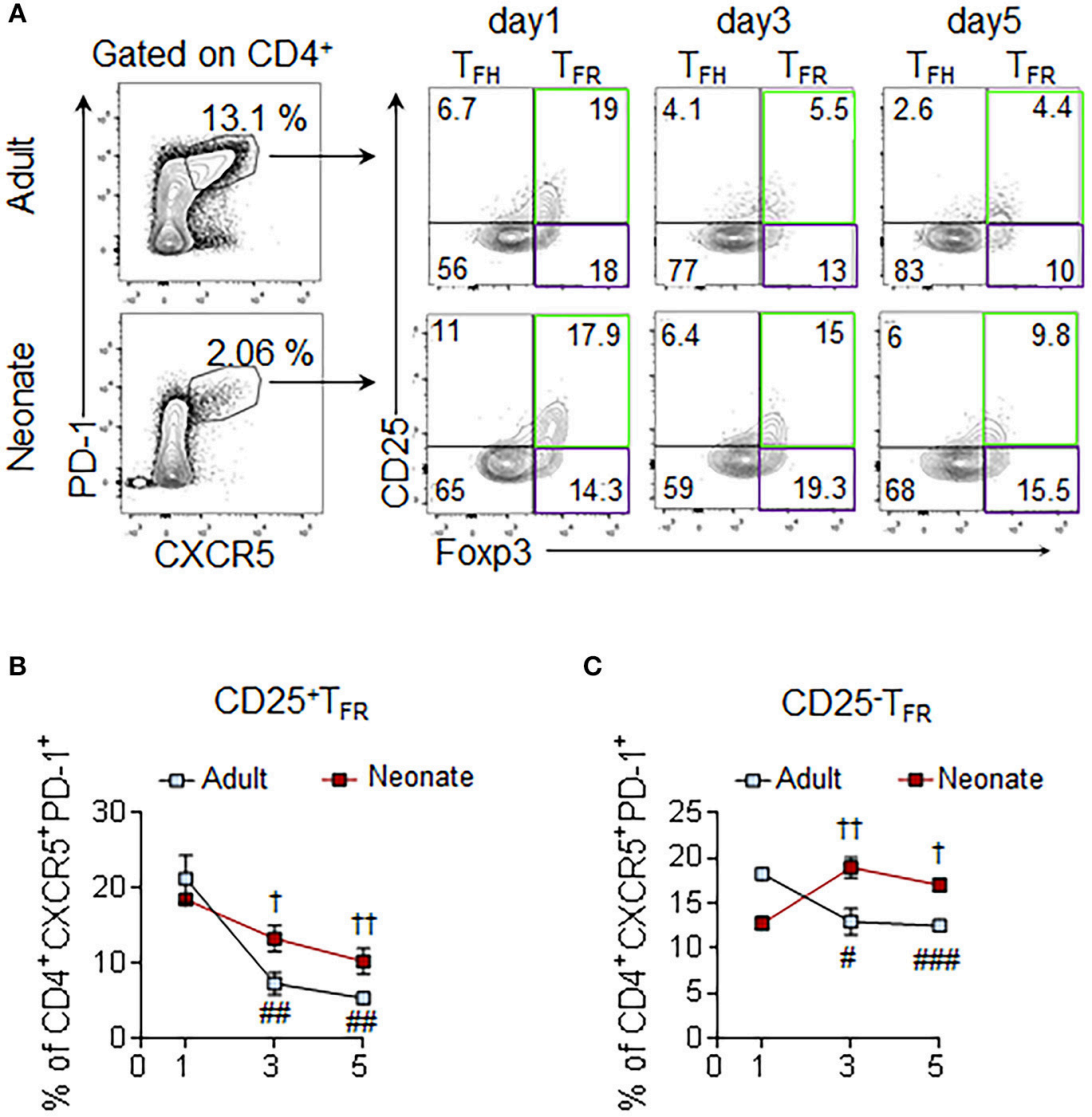
Assessment of CD25^+^ and CD25^−^ subsets of T_FR_ cells. Adult (6 to 10-week-old) and neonatal (5 day-old) mice were immunized i.p. with SRBC. Splenic CD4^+^ cells were pre-gated prior to the analysis of CD25^+^ and CD25^−^ T_FR_ (PD-1^+^CXCR5^+^Foxp3^+^) subsets. **(A)** Representative dot plots depict the percentage of CD25^+^ (green gate) and CD25^−^ (purple gate) T_FR_ cells on 1, 3, and 5 dpi. Mean percentages of CD25^+^ T_FR_ **(B)** and CD25^−^ T_FR_ **(C)** cells among CD4^+^CXCR5^+^PD-1^+^ cells are plotted (*n* = 4). Error bar, s.e.m.. ^#^*p* < 0.05, ^*##*^*p* < 0.01, ^*###*^*p* < 0.001 for 1 dpi vs. 3 dpi or 5 dpi of adult mice. ^†^*p* < 0.05, ^††^*p* < 0.01 for 1 dpi vs. 3 dpi or 5 dpi of neonatal mice.

The flow cytometry and immunohistochemistry results suggested that the neonatal T_FR:_ T_FH_ ratio is higher than the adult mouse ratio and more Foxp3 cells occupy the neonatal follicules than the adult follicules. More detailed characterization of the T_FR_ cells by three different groups recently demonstrated that despite originating from CD25^hi^ T_reg_ cells, the T_FH_-suppressive T_FR_ cells occupying the follicules do not express CD25 (36–38). To determine the ratio of these two T_FR_ subsets in the follicules, we quantified the Foxp3-expressing CD25^+^ and CD25^−^ T_FR_ cells among the CD4^+^CXCR5^+^PD-1^+^ population. In immunized adult mice, both CD25^+^ T_FR_ and CD25^−^ T_FR_ decreased steadily after 1 dpi (Figures 5A–C). In sharp contrast, neonatal mice CD25^−^ T_FR_ cells increased significantly after 1 dpi. Thus, not only immunization elicited higher T_FR:_ T_FH_ ratio in neonatal mice than adult mice, but also the frequency of T_FR_ cells co-occupying the follicules with T_FH_ cells increased following immunization.

### Profound Defect in IL-21 Signaling on Neonatal GC B Cells

**Figure 6:**
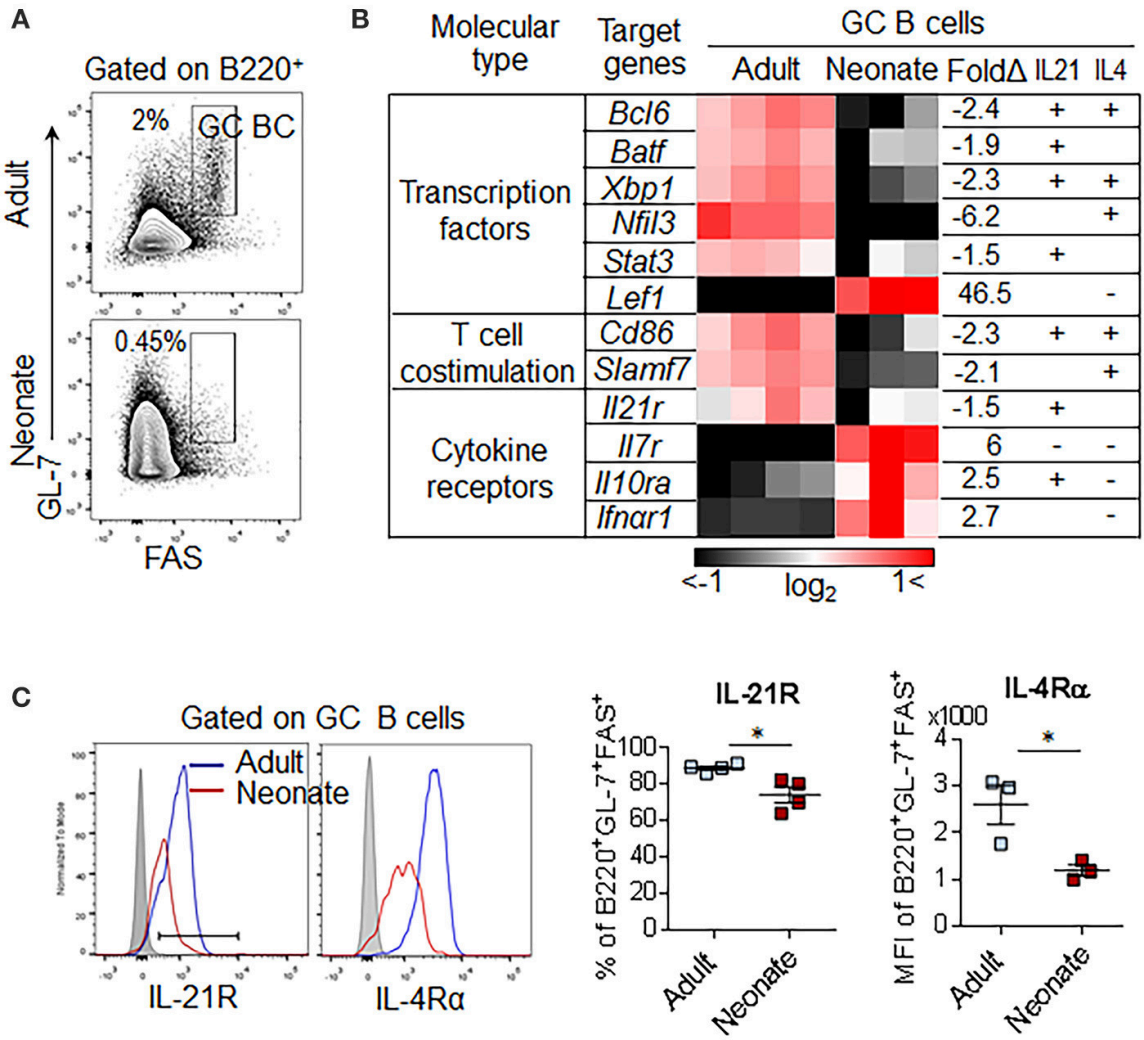
GC B cell pathway analysis and receptor expression. Adult (6 to 10-week-old) and neonatal (5 day-old) mice were immunized i.p. with SRBC. **(A)** Representative gating of GC B (B220^+^GL-7^+^FAS^+^) 10 dpi for transcriptional analysis. Plots were pre-gated on B220^+^ B cells. **(B)** Comparison of GC B cell genes found in ingenuity pathway upstream analysis among genes differentially expressed in neonatal vs. adult GC B cells (*p* < 0.05, >1.5-fold). Selected genes were predicted as downstream genes induced by IL-21 and IL-4. Each column represents one replicate pooled from three adult mice or eight neonatal mice. Upregulated genes, induced by either IL-21 or IL-4 were marked (+) and downregulated genes were marked (-). **(C)** Flow cytometry of splenocytes from immunized mice at 7dpi. Plots were pre-gated on B220^+^GL-7^+^FAS^+^ GC B cells (*n* = 3–4). Error bar, s.e.m.. *p < 0.05.

Our analysis of immunized neonatal mice has shown that GC B cell development is severely impaired (Figure 1E). In adults, the response of GC B cells is controlled by IL-21 and IL-4 secreted from T_FH_ cells (39). To gain insight into the differences in transcriptional programs elicited by these cytokines, we analyzed the expression profiles of genes known to be controlled by these cytokines in sorted B220^+^FAS^+^GL-7^+^ GC B cells from adult and neonatal mice (Figure 6A). Ingenuity upstream pathway analysis of transcriptional signatures in GC B cells (>1.5-fold, *p* < 0.05 adult vs. neonatal GC B cells) indicated that the expression of many genes, including those encoding the essential GC B cell transcription factors (*Bcl6, Batf, Xbp1*, and *Nfil3*) and T cell co-stimulatory molecules (*Cd86* and *Slamf7)* were not induced in neonatal GC B cells (Figure 6B). These genes are known to be induced or suppressed by IL-21 and/or IL-4, as marked “+ or –,” respectively. The large number of important transcription factors and signaling molecules that were weakly expressed in neonatal GC B cells are downstream targets of IL-21. This analysis confirmed the profound defect in IL-21 signaling on neonatal GC B cells. Interestingly, *in vitro* stimulation experiments suggested that splenic neonatal B cells are capable of inducing the expression of Bcl6 at levels comparable to those of adult B cells in response to IL-21, CD40L, and CpG (40, 41) (Supplementary Figures 3A–C). However, severely reduced expression of IL-21 by neonatal CD4^+^PD-1^+^ cells (Figure 1D and Supplementary Figure 1) together with the dampened expression of IL-4Rα and IL-21R on neonatal GC B cells (Figure 6C) likely favored a blunted GC B cell response in immunized neonatal mice.

## Discussion

**Figure 7:**
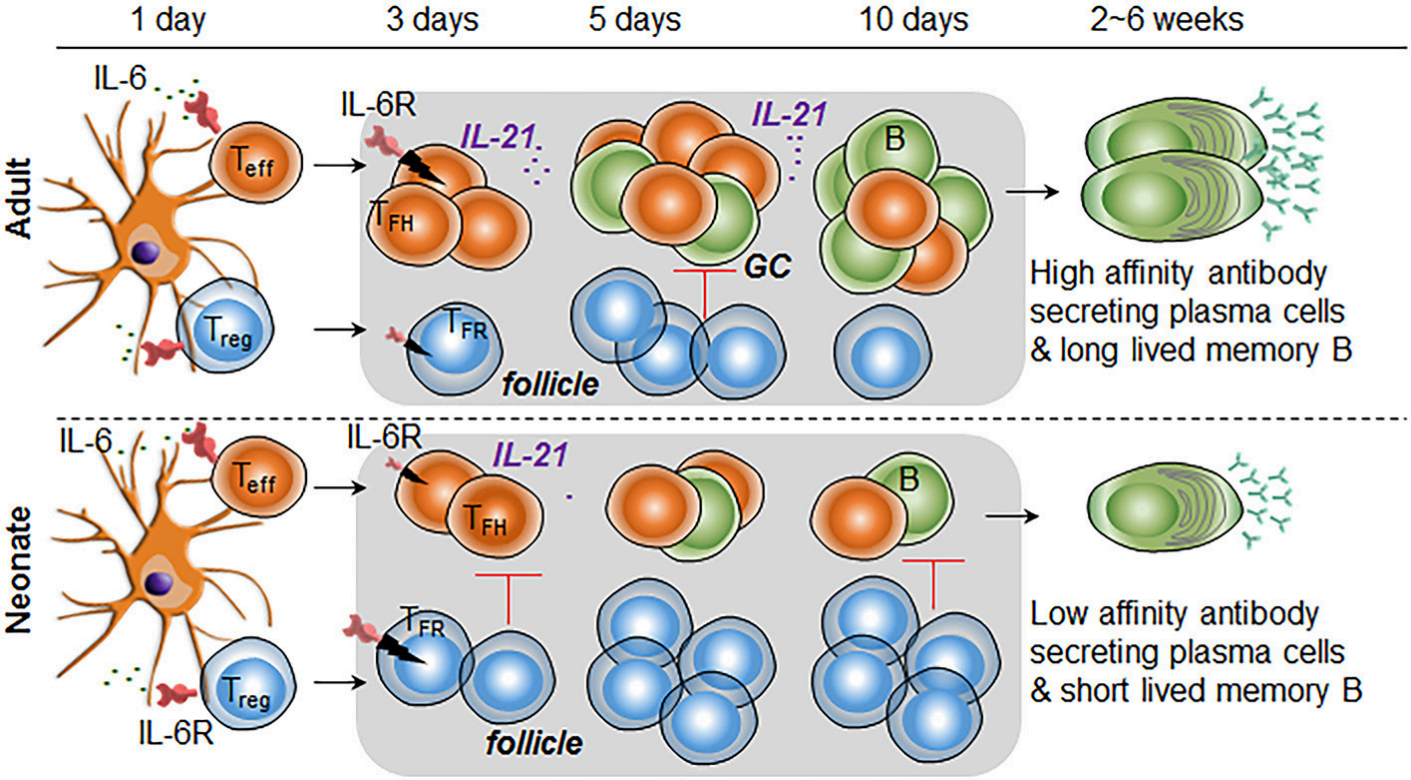
Higher IL-6R expression on T_FR_ cells than T_FH_ cells is responsible for increased T_FR:_ T_FH_ ratio in immunized neonatal mice. In immunized neonatal mice, IL-6 (likely secreted from antigen presenting DCs) is unable to expand T_FH_ cells because of diminished signaling, a likely consequence of lower IL-6R expression. In addition, elevated IL-6 activity in T_FR_ cells leads to the expansion of this subset in neonates. Consequently, in response to IL-6, T_FR_ cells populate the follicles and suppress the expansion of IL-21 expressing T_FH_ cells and GC B cells. Diminished T_FH_ and GC B cell formation leads to low number of plasma cell and short-lived memory cell development.

Recent discovery of impaired T_FH_ cell-development accompanied by the predominance of the inhibitory regulatory T cells in immunized neonatal mice provided fresh insights into the weak vaccine responses in neonatal period (15, 16). We undertook this study to explore the immunobiological mechanisms responsible for the impaired T_FH_ cell generation in immunized neonates. We focused on the mediators of T_FH_ cell-expansion following immunization and found limited activity of IL-6 on neonatal T_FH_ cells, a cytokine that is essential in the production of IL-21 and expansion of T_FH_ cells in adult mice (21, 24–26). Consequently, inclusion of IL-6 in PPS14-TT conjugate vaccine suppressed the generation of T_FH_ cells and antibody responses (Figure 7). We also determined that reduced expression of IL-6R complex on T_FH_ cells is likely responsible for the ablated production of IL-21 and expansion of T_FH_ cells.

In the lymphoid organs, IL-6 is expressed by antigen-presenting cells (APC) such as DC, macrophages, and B cells; while IL-6R is expressed on APC and T cells (28). IL-6 plays an important modulatory role for CD4^+^ T cell effector function during TD responses. DC-derived IL-6 promotes the initiation of the T_FH_ differentiation program following immunization (20, 26). In addition to aiding in early T_FH_ development, IL-6 produced by follicular DCs as well as B cells is critically important in B cell responses during the later stages of GC reaction (42–44). Underscoring the impact of IL-6 in TD responses, injection of IL-6 with an inactivated influenza virus is shown to boost IL-21 mediated GC responses accompanied by augmented antibody development (21). An important distinction between adult and neonatal innate cells is the elevated production of IL-6 from neonatal leukocytes as compared to adult cells in response to stimulation with TLR agonists (45). Yet, little is known about the impact of heightened IL-6 production in neonatal vaccine responses. Here, we demonstrated that despite the propensity to produce more IL-6 from APC (45), IL-6 induced STAT3 phosphorylation in neonatal T_FH_ cells was reduced compared to those of adult cells, a likely consequence of the reduced expression of the GP130 component of IL-6R complex on neonatal T_FH_ cells. The higher STAT3 phosphorylation observed in adult cells are in support of the report by Dienz et al. who have shown that co-injection of adult mice with 500 ng of IL-6 with an influenza vaccine elicited higher antibodies than mice injected with influenza vaccine alone (21). Although Dienz et. al. did not measure T_FH_ response in IL-6 co-injected mice, the fact that IL-6 mediated augmentation of antibody production was dependent on IL-21 suggests that the beneficial effect of IL-6 involves T_FH_ expansion. Using the same amount of IL-6, we were able confirm the adjuvant activity of IL-6 for PPS14-TT vaccine in adult mice, which manifested higher T_FH_ and GC response. Unlike its stimulatory effect in adult mice, the injection of weight adjusted amount of IL-6 (100 ng) in neonatal mice led to suppression of IgG and IgA antibody production. Moreover, the decrease in neonatal antibody responses was accompanied by reduced T_FH_ cell development. The inability of IL-6 to increase T_FH_ cell response could be explained by the reduced STAT3 phosphorylation in T_FH_ cells following IL-6 stimulation but the significant reduction in T_FH_ and antibody responses compared to neonates immunized without the IL-6 containing PPS14-TT vaccine suggested an IL-6 mediated suppression in addition to blunted T_FH_ response. In support of this hypothesis, we measured higher frequency and numbers of the suppressive T_FR_ cells in IL-6 co-injected mice compared to those immunized with the vaccine alone. *In vitro* signaling experiments provided an explanation for the increase in T_FR_ cells after IL-6 injection because we found increased STAT3 phosphorylation in neonatal T_FR_ cells than adult cells in response to IL-6 stimulation, a likely consequence of higher expression of IL-6R on neonatal T_FR_ cells than adult cells (Figure 7). Additionally, the localization of higher numbers of Foxp3^+^ cells in the B cell zone in immunohistochemistry and the measurement of higher numbers of CD25^−^ T_FR_ cells that govern the GC B cells (36–38) in immunized neonatal mice than the adult mice collectively suggest an environment that favors suppressed T_FH_ responses.

The factors that regulate IL-6R complex expression on T_FH_ and T_FR_ cells are poorly understood. Interestingly, unlike CD25 that is upregulated by TCR stimulation in activated CD4^+^ T cells (46), IL-6Rα level is reduced during adult mice immunization (28). For example, *in vitro* TCR and costimulatory molecule signaling induces IL-2 expression, and subsequent IL-2 signaling increases CD25 expression through a STAT5-dependent positive feedback loop (46). In contrast, TCR and costimulatory molecule signaling reduce IL-6Rα expression on CD4^+^ T cells (47), likely by inducing adamalysins ADAM17 and ADAM10, which are proteases responsible for the shedding of IL-6Rα (48). Like the IL-6Rα expression, GP130 expression can be downregulated by TCR stimulation (47). In addition, p38 activation through inflammatory cytokines such as IL-1β or TNF-α can lead to GP130 internalization and degradation (49). The requirement for CD28 and ICOS for the generation of T_FH_ and T_FR_ cells suggests the need for interactions at the T-cell zone with DCs (6, 14). The inability of neonatal T_FR_ cells to decrease IL-6Rα expression despite elevated STAT3 phosphorylation or the insufficient GP130 expression on T_FH_ cells may be associated with suboptimal DC priming of naïve CD4^+^ T cells in the extrafollicular area. The subsets, as well as the functions of DCs show important differences between neonates and adults. In addition to the reported deficiencies in antigen uptake, MHCII, CD80, CD86, and CD40 expression/function (50), potential differences in PD-L1 and ICOSL expression/function may contribute to the persistence of IL-6Rα in neonatal T_FR_ cells (51).

The suppressive environment in neonatal lymphoid organs was not limited to T_FH_ cell development. We also found lower levels of IL-21R and IL-4R in immunized neonatal mice GC B cells. Both IL-21 and IL-4 promote plasma cell development from B cells (52, 53). IL-21R expression level is low in immature B cell subset, newly-migrated T1 B cells and marginal zone B cells, whereas its level is high in mature B cell subsets, T2 B cells and follicular B cells (52). Since most B cells of neonatal mice are immature T1 B cells, low GC B cell-IL-21R expression is somewhat expected (4). Nevertheless, neonatal B cells were not intrinsically restricted to differentiate into GC B cells because *in vitro* stimulation of B cells with IL-21 and CD40L or CpG induced comparable levels of Bcl6 expression in neonatal and adult B cells. Thus, both the production of IL-21 by T_FH_ cells and the lower expression of IL-21R and IL-4R on GC B cells emerge as limiting factors for plasma cell differentiation in immunized neonatal mice.

Together, we unveiled a previously undefined mechanism of limited T_FH_ differentiation in neonates. We found that combined with enhanced signaling in T_FR_ cells, diminished response to IL-6 in T_FH_ cells likely govern the weak antibody responses to vaccines in neonates. Our findings provide fresh insight into different mechanisms for GC programing of IL-6 signaling in adult and neonates. We documented a detrimental effect of IL-6 co-injection in the development of antibody responses against a TD vaccine, a strategy proposed to boost vaccine responses based on findings in adult mice (21). While this difference provides opportunities to improve infant vaccines by using adjuvants that can modulate IL-6R expression on T_FH_ and T_FR_ cells, it also highlights the unique features of the neonatal immune system and the importance of corroborating adult results in neonates before translating them to other age groups.

## Author Contributions

MA designed experiments, analyzed data, created figures, and wrote/edited the manuscript. JY designed and executed the experiments, analyzed the data, created the figures, and wrote/edited the manuscript. JS, SS, and RL performed experiments. DI, and DV analyzed the data. MA conceived and directed the project, designed experiments, analyzed data, and edited the manuscript. All authors critically read and approved the manuscript.

### Conflict of Interest Statement

The authors declare that the research was conducted in the absence of any commercial or financial relationships that could be construed as a potential conflict of interest.
