# Supplementary material for: IL-6 Impairs Vaccine Responses in Neonatal Mice
Source: Front Immunol. 2018 Dec 20;9:3049. doi: 10.3389/fimmu.2018.03049 (PMC6307459; doi:10.3389/fimmu.2018.03049)
Supplement: Supplementary file 1 [file Data_Sheet_1.docx]

Supplementary figures

**IL-6 impairs vaccine responses in neonatal mice**
 
Jiyeon Yang, Jiro Sakai, Shafiuddin Siddiqui, Robert C Lee, Derek DC Ireland, Daniela Verthelyi, and Mustafa Akkoyunlu

**Supplementary Figure 1**

**

*

ns

ns

1.3


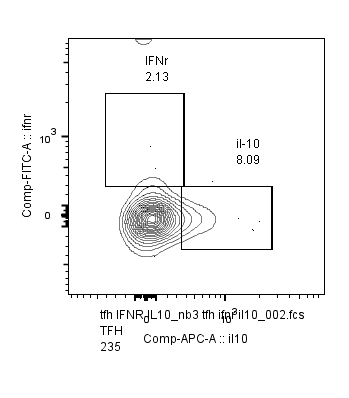


8.1

2.2


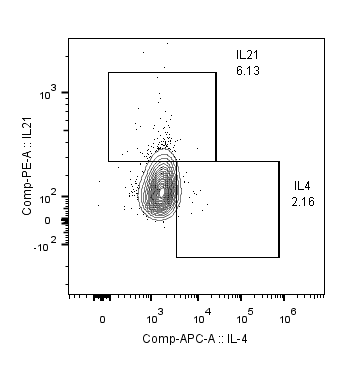


2.3

Neonate

Adult

6


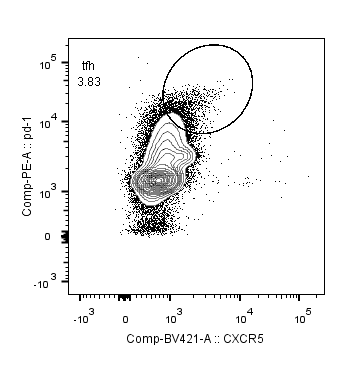

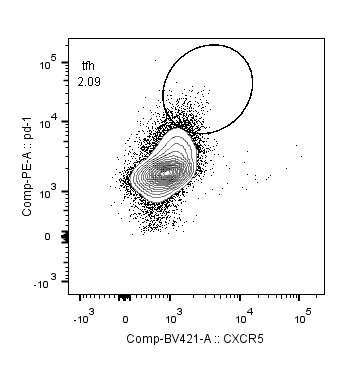

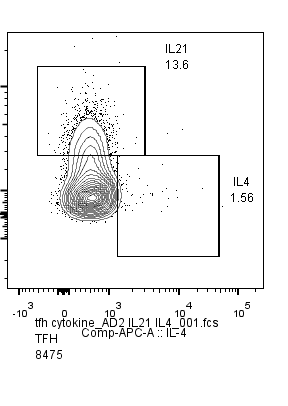


1.8

3.8

14


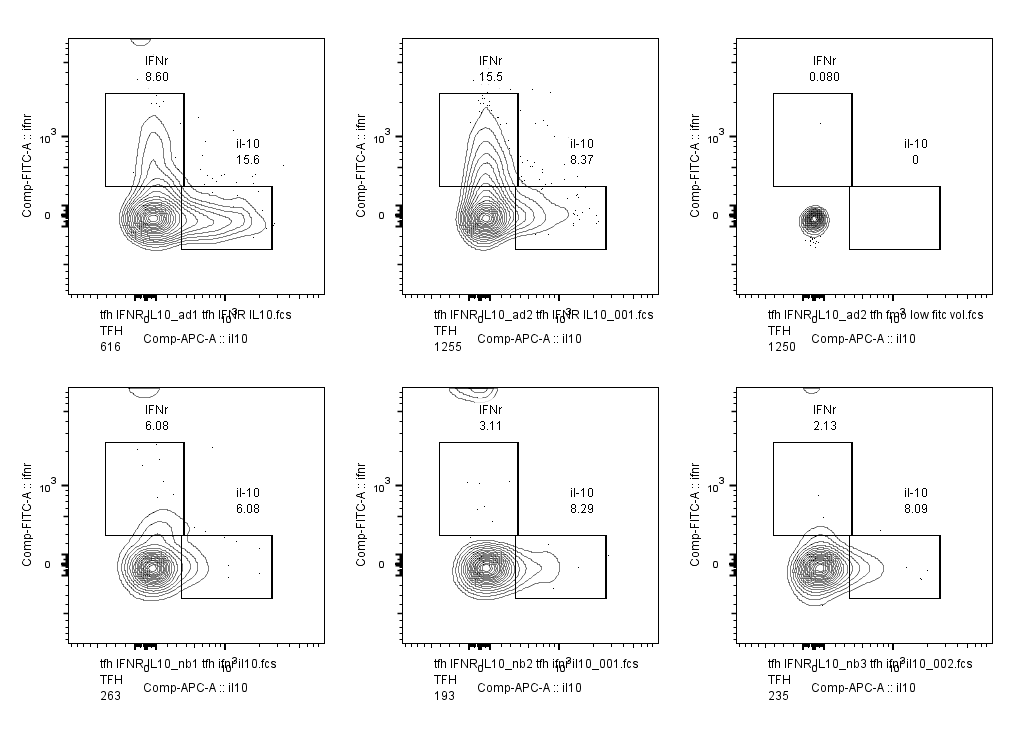


8.37

15.5

1.6

CXCR5

PD-1

IL-10

IFNγ

IL-4

IL-21

3.83

2

Gated on CD4

**Supplementary Figure 1. Cytokine secretion from ex vivo stimulated neonatal T helper cells.** Adult (6 to 10-week-old) and neonatal (5-day-old) mice were immunized i.p. with SRBC. Splenocytes were stimulated with PMA and ionomycin, followed by intracellular cytokine staining at 7 dpi (n=4). Error bar, s.e.m.. *p<0.05, **p<0.01 for adult vs neonatal mice.

**Supplementary Figure 2**


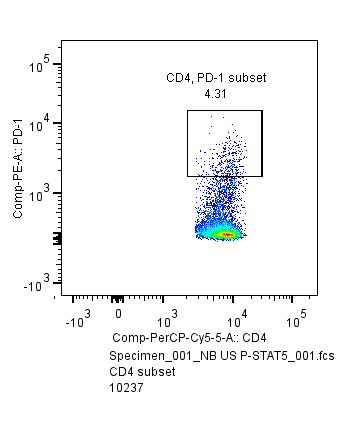

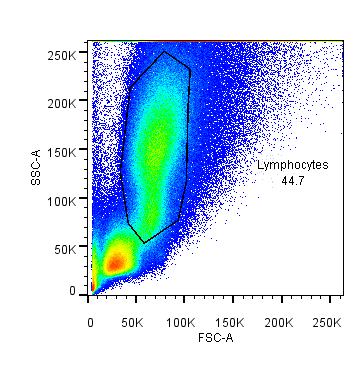


**Neonate**

PD-1

CD4

PD-1

CD4

SSC-H

FSC-A

SSC-A

**Adult**


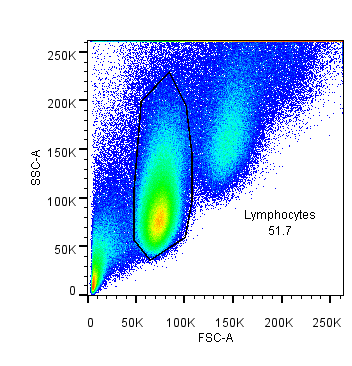

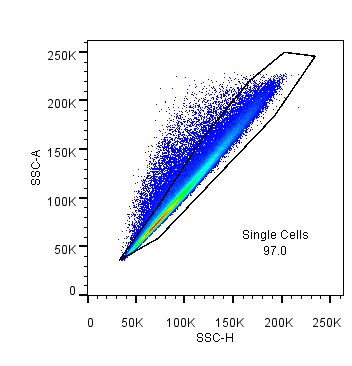

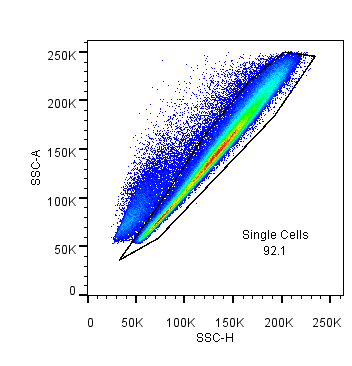

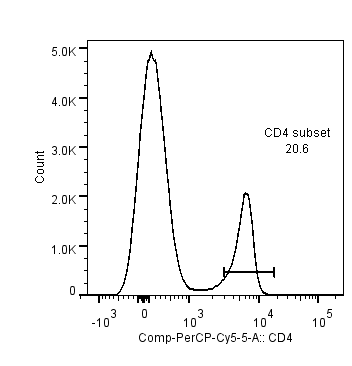

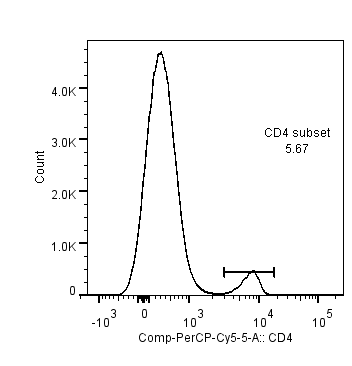

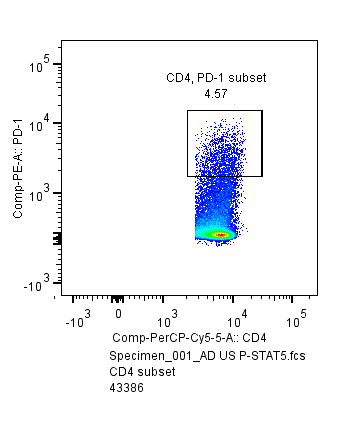


SSC-A

Lymphocytes

Single cells

CD4

Lymphocytes

Single cells

CD4

SSC-H

FSC-A

4.6

4.3

20.8

6

97

92

52

54

**Supplementary Figure 2. Pre-gating strategy for intracellular phospho-flow staining and CD25 expression levels on T_FH_ and T_FR_ populations.** Adult (6 to 10-week-old) and neonatal (5-day-old) mice were immunized i.p. with SRBC. Splenocytes from 5 dpi were gated on CD4^+^PD-1^hi^ cells prior to intracellular phospho-flow staining (STAT3) in flow cytometry.

**Supplementary Figure 3**

**
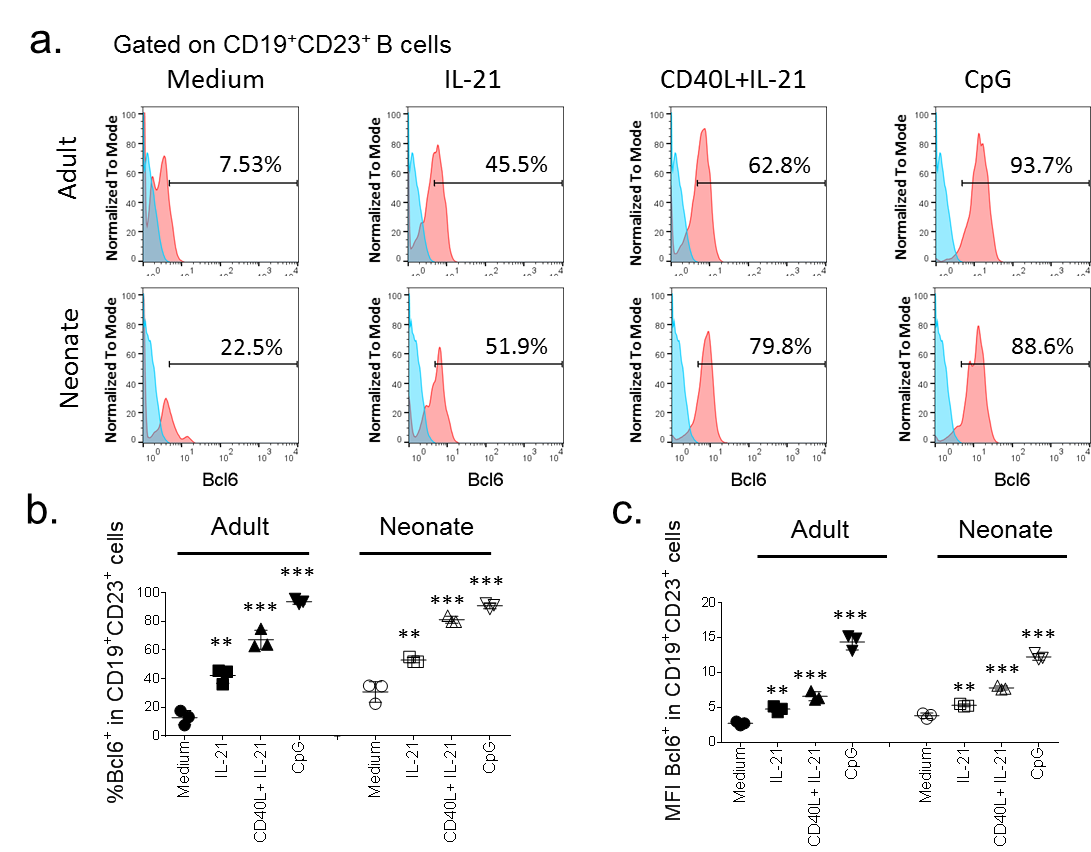
**

**Supplementary Figure 3. Bcl6 expression in stimulated B cells.** Splenic B cells from naïve neonatal (7-day-old) or adult (6 to 10-week-old) mice were cultured in medium, IL-21 (10 ng/ml), IL-21 and CD40L (1 μg/ml) or CpG (25 µg/ml) for 72 hours. **a.** Representative plots depict Bcl6 expression in CD19^+^CD23^+^ B cells. **b.** Average frequency and MFI **(c)** of Bcl6 expression on CD19^+^CD23^+^ cells are plotted. Error bar, s.e.m.. *p<0.05, **p<0.01, and ***p<0.001 for differences between media vs stimulated cells.
